# HGCLAMIR: Hypergraph contrastive learning with attention mechanism and integrated multi-view representation for predicting miRNA-disease associations

**DOI:** 10.1371/journal.pcbi.1011927

**Published:** 2024-04-23

**Authors:** Dong Ouyang, Yong Liang, Jinfeng Wang, Le Li, Ning Ai, Junning Feng, Shanghui Lu, Shuilin Liao, Xiaoying Liu, Shengli Xie

**Affiliations:** 1 Peng Cheng Laboratory, Shenzhen, China; 2 School of Biomedical Engineering, Guangdong Medical University, Dongguan, China; 3 Pazhou Laboratory (Huangpu), Guangzhou, China; 4 College of Mathematics and Informatics, South China Agricultural University, Guangzhou, China; 5 School of Computer Science and Engineering, Faculty of Innovation Engineering, Macau University of Science and Technology, Macau, China; 6 Computer Engineering Technical College, Guangdong Polytechnic of Science and Technology, Zhuhai, China; 7 Guangdong-HongKong-Macao Joint Laboratory for Smart Discrete Manufacturing, Guangzhou, China; Universidad de Costa Rica, COSTA RICA

## Abstract

Existing studies have shown that the abnormal expression of microRNAs (miRNAs) usually leads to the occurrence and development of human diseases. Identifying disease-related miRNAs contributes to studying the pathogenesis of diseases at the molecular level. As traditional biological experiments are time-consuming and expensive, computational methods have been used as an effective complement to infer the potential associations between miRNAs and diseases. However, most of the existing computational methods still face three main challenges: (i) learning of high-order relations; (ii) insufficient representation learning ability; (iii) importance learning and integration of multi-view embedding representation. To this end, we developed a HyperGraph Contrastive Learning with view-aware Attention Mechanism and Integrated multi-view Representation (HGCLAMIR) model to discover potential miRNA-disease associations. First, hypergraph convolutional network (HGCN) was utilized to capture high-order complex relations from hypergraphs related to miRNAs and diseases. Then, we combined HGCN with contrastive learning to improve and enhance the embedded representation learning ability of HGCN. Moreover, we introduced view-aware attention mechanism to adaptively weight the embedded representations of different views, thereby obtaining the importance of multi-view latent representations. Next, we innovatively proposed integrated representation learning to integrate the embedded representation information of multiple views for obtaining more reasonable embedding information. Finally, the integrated representation information was fed into a neural network-based matrix completion method to perform miRNA-disease association prediction. Experimental results on the cross-validation set and independent test set indicated that HGCLAMIR can achieve better prediction performance than other baseline models. Furthermore, the results of case studies and enrichment analysis further demonstrated the accuracy of HGCLAMIR and unconfirmed potential associations had biological significance.

## Author summary

Considerable studies have demonstrated that the dysregulation of miRNAs is closely related to human diseases. Therefore, inferring unconfirmed associations between miRNAs and diseases is helpful for disease diagnosis and treatment. Numerous computational models have been proposed to discover potential miRNA-disease associations on a large scale, which can accelerate the understanding of disease pathogenesis. We constructed a HGCLAMIR model to identify miRNA-disease associations through hypergraph convolutional network with contrastive learning, view-aware attention mechanism and integrated representation learning. The 5-fold cross-validation and independent testing were performed to evaluate the performance of HGCLAMIR, which was better than ten baseline models. In addition, we carried out case studies on breast neoplasms and lung neoplasms, showing that 49 and 48 of the top 50 candidate miRNAs were confirmed by experimental reports. In summary, HGCLAMIR could be considered as an effective and accurate model for predicting the associations between miRNAs and diseases.

## Introduction

MicroRNAs (miRNAs) are a class of single-stranded non-coding RNA molecules with a length of about 22 nucleotides, which play an important role in many biological processes by targeting mRNA [1–3]. To be more specific, miRNAs inhibit the translation of target mRNAs to prevent protein production or downregulate mRNA expression by binding to the 3’UTR of target mRNAs [4, 5]. Existing studies have shown that the overexpression or dysregulation of miRNAs may lead to the occurrence and development of various human diseases [6]. For example, the expression level of hsa-let-7 is significantly reduced in lung cancer, which verifies that miRNAs are closely related to tumors [7]. Studies have shown that hsa-mir-155 is identified as a candidate biomarker for early pancreatic tumors [8]. In addition, the expression of hsa-mir-18a in pancreatic cancer tissues and cell lines is significantly higher than in normal tissues [9]. Therefore, miRNAs may be potential biomarkers of various diseases, and further exploring the associations between miRNAs and diseases is of great significance for understanding the pathogenic mechanism at the molecular level. Traditional wet experiments can infer the associations between miRNAs and diseases, but they are time-consuming and expensive. Hence, computational methods, due to time-saving, cost-minimizing and large-scale discovery of potential associations, have been increasingly used as complementary tools to identify potential associations between miRNAs and diseases.

The existing computation-based methods for predicting the associations between miRNAs and diseases are mainly divided into two categories [10]. The first category is similarity measure-based methods, the basic assumption of which is that miRNAs with similar functions are more likely to be associated with diseases with similar phenotypes, and vice versa. Chen *et al*. [11] developed the RWRMDA method to infer potential miRNA-disease associations by implementing random walks on miRNA-miRNA functional similarity networks. Chen *et al*. [12] integrated known miRNA-disease associations, miRNA functional similarity network, disease semantic similarity network and Gaussian interaction profile kernel similarity network, and developed the model of Within and Between Score for MiRNA-Disease Association prediction (WBSMDA) based on the integrated similarity. You *et al*. [13] proposed a path-based search method PBMDA, which uses a depth-first search strategy to predict the associations between miRNAs and diseases. Chen *et al*. [14] proposed a computational model of Bipartite Network Projection for MiRNA–Disease Association prediction (BNPMDA) based on the bias ratings by exploiting the integrated similarity related to miRNAs and diseases. Chen *et al*. [15] designed a bipartite heterogeneous network association prediction method based on co-neighbor to predict miRNA-disease associations. Zhang *et al*. [16] presented a fast linear neighborhood similarity-based network method called FLNSNLI to predict miRNA-disease associations. The second category is machine learning-based methods. As more and more associations between miRNAs and diseases have been confirmed by biological experiments, it is possible to predict potential miRNA-disease associations in a data-driven manner. Fu *et al*. [17] utilized a stacked autoencoder to extract the embedding representations of nodes from miRNA and disease similarity networks as feature vectors for miRNA-disease pairs, and input them into a three-layer neural network to predict miRNA-disease associations. Chen *et al*. [18] proposed the RFMDA method combining filter-based feature selection strategy and random forest classifier to predict the associations between miRNAs and diseases. Chen *et al*. [19] presented a prediction model called EGBMMDA based on extreme gradient boosting for miRNA-disease association prediction. Ji *et al*. [20] developed a deep autoencoder-based computational method named AEMDA, which can extract embedding representations of diseases and miRNAs from similarity matrices for predicting the associations between miRNAs and diseases. Liu *et al*. [21] proposed a computational method called SMALF. It uses a stacked autoencoder to learn miRNA and disease embedding representations, and predicts unknown miRNA-disease associations based on eXtreme Gradient Boosting (XGBoost). Dong *et al*. [22] developed a biologically-motivated data-driven method called MPM to identify miRNA-disease associations. MPM applies a message passing framework to enrich existing biological associations and uses a random forest classifier to predict the miRNA-disease association probabilities.

In recent years, graph-based neural networks can effectively aggregate information between nodes through message passing in graph-structured data, which has demonstrated powerful feature representation ability. Tang *et al*. [23] used graph convolutional network (GCN) and attention mechanism to extract and enhance latent representations of miRNAs and diseases, and predict potential associations based on the reconstructed miRNA-disease association matrix. Dong *et al*. [24] proposed a multi-task graph convolutional learning framework named MuCoMiD, which integrates knowledge from five heterogeneous biological information sources and allows automatic feature extraction in an end-to-end manner to predict the associations between miRNAs and diseases. Wang *et al*. [25] designed the MAGCN method based on known lncRNA–miRNA interactions and graph convolution networks without using any similarity measurements. This method predicts miRNA-disease associations by using GCN with multichannel attention mechanism and convolutional neural network combiner. Ruan *et al*. [26] developed the MSGCL method to optimize the graph structure by applying self-supervised contrastive learning, which uses a graph convolutional network encoder to identify the associations between miRNAs and diseases. Nevertheless, these graph-based neural network methods usually represent the relationship between nodes as a bipartite graph, which results in only aggregating the information of neighbor nodes and failing to learn high-order relations. Hypergraphs, as an extension of bipartite graphs, utilize a subset of nodes as a hyperedge, thereby effectively capturing high-order relations between nodes. Wu *et al*. [27] designed an MSCHLMDA method of multi-similarity based on combinative hypergraph learning for predicting miRNA-disease associations, which makes use of K-nearest neighbor (KNN) and K-means methods to construct two different hypergraphs. Wang *et al*. [28] presented the HFHLMDA method to infer the miRNA-disease associations based on high-dimensionality features and hypergraph learning. HFHLMDA can effectively learn the high-order relations among miRNA-disease pairs by applying hypergraph Laplacian regularization on the projection matrix. However, these methods are unable to learn nonlinear feature representations related to miRNAs and diseases, which limits the improvement of prediction performance. To learn high-order relations while capturing nonlinear information, Ning *et al*. [29] developed a method called AMHMDA based on attention aware multi-view similarity networks and hypergraph learning. This method introduces hypernodes in graph convolution network to learn high-quality links and richer node information for miRNA-disease association identification. However, AMHMDA still uses standard GCN and lacks effective strategies to further enhance embedding representation learning ability, resulting in suboptimal prediction results.

Although all the above methods have achieved excellent performance in discovering potential associations, most of them still have some limitations. On the one hand, similarity measure-based methods rely too much on known association information, which leads to poor performance on association prediction for new or rare diseases. On the other hand, the quality of the embedded representations of miRNAs and diseases has a critical impact on the performance of association prediction. Although some models based on graph convolutional networks have been proposed to learn high-quality nonlinear embedding representations [23, 30], they only focus on pairwise relations in homogeneous graphs and ignore high-order complex relations in heterogeneous graphs. In addition, existing graph-based methods have the problem of insufficient learning ability of embedding representations. Moreover, these methods ignore the importance of different views and the degree of concern between different views, thus affecting the quality of integrated miRNA or disease embedding representation.

To alleviate the abovementioned limitations, we proposed **H**yper**G**raph **C**ontrastive **L**earning with view-aware **A**ttention **M**echanism and **I**ntegrated multi-view **R**epresentation, named HGCLAMIR, for miRNA-disease association prediction. First, we used KNN and K-means methods to construct hypergraphs of two different views of miRNAs (or diseases) from a miRNA-disease heterogeneous network, respectively. Then, hypergraph convolutional network (HGCN) was employed to capture high-order complex relations from hypergraphs related to miRNAs or diseases. Next, we combined HGCN with contrastive learning to improve and enhance the embedded representation learning ability of HGCN, thereby learning more higher quality embedding representation information. Moreover, view-aware attention mechanism was introduced to adaptively weight the embedded representations of different views for obtaining the importance of multi-view latent representations. To obtain more richer and reasonable embedding information, we innovatively proposed integrated representation learning to integrate two-view embedding representations of miRNAs or diseases. Finally, we utilized a neural network-based matrix completion method to predict miRNA-disease associations based on integrated embedding information. Meanwhile, we conducted extensive experiments to evaluate the prediction performance of our model on two different datasets. The experimental results of 5-fold cross-validation five times and independent testing indicated that HGCLAMIR was better than other baseline models. In addition, the results of ablation studies demonstrated the effectiveness of each module of our model. Furthermore, the results of case studies further confirmed that HGCLAMIR can accurately predict the associations between miRNAs and diseases, as well as unconfirmed potential miRNA-disease associations had biological significance. In conclusion, HGCLAMIR can be used as an effective tool to discover potential miRNA-disease associations.

## Materials and methods

### Human miRNA-disease associations

Since the miRNA-disease associations in Human MiRNA Disease Database (HMDD) have been experimentally verified, the HMDD database was often used for miRNA-disease association research [31, 32]. In this paper, we generated two miRNA-disease association datasets from HMDD v2.0 and HMDD v3.2 database, where these two databases can be downloaded from https://www.cuilab.cn/hmdd. The first dataset MDAv2.0 includes 5425 experimentally verified associations between 380 diseases and 495 miRNAs, whereas the second dataset MDAv3.2 contains 486 diseases and 917 miRNAs, and provides 9732 experimentally verified human miRNA-disease associations. Next, we can construct adjacency matrix *T* ∈ {0, 1}^*M*×*D*^ with 0–1 entries based on the HMDD database, where *M* and *D* represent the number of miRNAs and diseases, respectively. The adjacent matrix *T* indicates the known miRNA-disease associations, where *T*(*i*, *j*) = 1 if a miRNA *i* is associated with a disease *j*, *T*(*i*, *j*) = 0 if the association between a miRNA *i* and a disease *j* is unknown or unobserved.

### Disease semantic similarity

The disease descriptors were utilized to calculate disease semantic similarity [33], which can be obtained from the Medical Subject Headings (MeSH) database (https://www.nlm.nih.gov/mesh/). To be more specific, the Directed Acyclic Graph (DAG) can be used to describe the hierarchical relationships of different diseases. For a disease *d*_*i*_, we defined *DAG*(*d*_*i*_) = (*d*_*i*_, *T*(*d*_*i*_), *E*(*d*_*i*_)), where *T*(*d*_*i*_) represents a set of nodes including *d*_*i*_ itself and its ancestor nodes, *E*(*d*_*i*_) denotes the edge set with regard to the direct links between the parent nodes and the child nodes. Then, the semantic contribution of diseases *d*_*k*_ to *d*_*i*_ can be calculated as follows:

$$\begin{array}{c} \begin{array}{cc} D1(d_{i},d_{k}) & =\{\begin{array}{cc} 1, & \text{if}\;d_{k}=d_{i}\\ max\{\Delta \times D1\left(d_{i},d_{k}^{\prime }\right)|\\ d_{k}^{\prime }\in children\;of\;d_{k}\}, & \text{if}\;d_{k}\neq d_{i}. \end{array} \end{array} \end{array}$$ (1)

where Δ is a semantic contribution decay factor and it is set to 0.5 according to previous work [33]. Concretely, the semantic contribution value of disease *d*_*i*_ to itself is 1, and the semantic contribution value of disease *d*_*k*_ to disease *d*_*i*_ progressively decreases as the distance between them increases. Therefore, the semantic value of disease *d*_*i*_ can be formulated as below:

$$\begin{array}{c} SV1\left(d_{i}\right)=\sum_{d_{k}\in T\left(d_{i}\right)}D1\left(d_{i},d_{k}\right) \end{array}$$ (2)

Based on the assumption that if a disease pair shares a large part of DAGs, they can be considered to have higher similarity between them. Then, we can obtain the disease semantic similarity *DSS*1(*d*_*i*_, *d*_*j*_) between diseases *d*_*i*_ and *d*_*j*_ as follows:

$$\begin{array}{c} \begin{array}{c} DSS1\left(d_{i},d_{j}\right)=\frac{\sum_{d_{t}\in T\left(d_{i}\right)\cap T\left(d_{j}\right)}\left(D1\left(d_{i},d_{t}\right)+D1\left(d_{j},d_{t}\right)\right)}{SV1\left(d_{i}\right)+SV1\left(d_{j}\right)} \end{array} \end{array}$$ (3)

However, *DSS*1 ignores the importance of the semantic contributions of different diseases. Because diseases appearing in less DAGs may be more specific and should have higher semantic contribution values, the semantic contribution values of diseases in the same layer of DAGs should be different. Based on previous study [34], the second semantic contribution of disease *d*_*k*_ to *d*_*i*_ can be presented as below:

$$\begin{array}{c} \begin{array}{c} D2\left(d_{i},d_{k}\right)=-\text{log}\left(\frac{the\;number\;of\;DAGs\;including\;d_{k}}{the\;number\;of\;disease}\right) \end{array} \end{array}$$ (4)

Similarly, we can obtain the second semantic value *SV*2(*d*_*i*_) of disease *d*_*i*_ and the disease semantic similarity *DSS*2(*d*_*i*_, *d*_*j*_) between diseases *d*_*i*_ and *d*_*j*_ as follows:

$$\begin{array}{c} SV2\left(d_{i}\right)=\sum_{d_{k}\in T\left(d_{i}\right)}D2\left(d_{i},d_{k}\right) \end{array}$$ (5)

$$\begin{array}{c} \begin{array}{c} DSS2\left(d_{i},d_{j}\right)=\frac{\sum_{d_{t}\in T\left(d_{i}\right)\cap T\left(d_{j}\right)}\left(D2\left(d_{i},d_{t}\right)+D2\left(d_{j},d_{t}\right)\right)}{SV2\left(d_{i}\right)+SV2\left(d_{j}\right)} \end{array} \end{array}$$ (6)

To obtain a more reasonable disease semantic similarity, we integrated these two kinds of disease semantic similarity *DSS*1(*d*_*i*_, *d*_*j*_) and *DSS*2(*d*_*i*_, *d*_*j*_) on the basis of previous study [35]. Finally, the disease semantic similarity *DSS*(*d*_*i*_, *d*_*j*_) between diseases *d*_*i*_ and *d*_*j*_ can be presented according to the following equation:

$$\begin{array}{c} DSS\left(d_{i},d_{j}\right)=\frac{DSS1\left(d_{i},d_{j}\right)+DSS2\left(d_{i},d_{j}\right)}{2} \end{array}$$ (7)

### MiRNA functional similarity

Based on the assumption that miRNAs associated with similar diseases may have similar functions, the miRNA functional similarity score can be calculated according to disease semantic similarity [33]. Then, we can build a miRNA functional similarity matrix *MFS*. *MFS*(*m*_*i*_, *m*_*j*_) denotes each element in the matrix *MFS*, which also represents the miRNA functional similarity score between miRNAs *m*_*i*_ and *m*_*j*_. Finally, *MFS* can be calculated by the following formula:

$$\begin{array}{c} \begin{array}{c} MFS\left(m_{i},m_{j}\right)=\frac{\sum_{d\in D(m_{i})}\;DSS\left(d,d_{j}^{*}\right)\;+\;\sum_{d\in D(m_{j})}\;DSS\left(d,d_{i}^{*}\right)}{|D\left(m_{i}\right)|+|D\left(m_{j}\right)|} \end{array} \end{array}$$ (8)

where *D*(*m*_*i*_) denotes the set of diseases that are associated with *m*_*i*_, |*D*(*m*_*i*_)| represents the number of elements in the set *D*(*m*_*i*_) and $d_{i}^{*}=\underset{d_{i}\in D\left(m_{i}\right)}{argmax}DSS\left(d,d_{i}\right)$.

### Gaussian interaction profile kernel similarity for miRNAs and diseases

Since miRNAs with similar function are likely to be associated with diseases with similar phenotypes, the Gaussian interaction profile kernel similarity has been calculated to represent miRNA similarity and disease similarity in previous studies [35, 36]. For a given miRNA *m*_*i*_, a binary vector *IP*(*m*_*i*_) was extracted from the known miRNA-disease associations to represent associations between miRNA *m*_*i*_ and each disease. Then, the Gaussian interaction profile kernel similarity for miRNAs *GPSM*(*m*_*i*_, *m*_*j*_) between miRNAs *m*_*i*_ and *m*_*j*_ can be presented as follows:

$$\begin{array}{c} GPSM\left(m_{i},m_{j}\right)=\text{exp}(-\gamma_{m}∥IP\left(m_{i}\right)-IP\left(m_{j}\right){∥}^{2}) \end{array}$$ (9)

where the parameter *γ*_*m*_ controls the kernel bandwidth, which can be calculated by using the following equation:

$$\begin{array}{c} \gamma_{m}=\frac{\gamma_{m}^{\prime }}{\frac{1}{M}\sum_{i=1}^{M}{∥IP\left(m_{i}\right)∥}^{2}} \end{array}$$ (10)

where *M* represents the number of miRNAs. Here, $\gamma_{m}^{\prime }$ is set to 1 according to the previous work [36]. Similarly, the Gaussian interaction profile kernel similarity for diseases *GPSD*(*d*_*i*_, *d*_*j*_) between diseases *d*_*i*_ and *d*_*j*_ can be calculated based on the following two equations:

$$\begin{array}{c} GPSD\left(d_{i},d_{j}\right)=\text{exp}(-\gamma_{d}∥IP\left(d_{i}\right)-IP\left(d_{j}\right){∥}^{2}) \end{array}$$ (11)

$$\begin{array}{c} \gamma_{d}=\frac{\gamma_{d}^{\prime }}{\frac{1}{D}\sum_{i=1}^{D}{∥IP\left(d_{i}\right)∥}^{2}} \end{array}$$ (12)

where a binary vector *IP*(*d*_*i*_) represents whether a disease *d*_*i*_ is associated with each miRNA in the known miRNA-disease associations, *D* refers to the number of diseases and $\gamma_{d}^{\prime }$ is also set to 1.

### Integrated similarity for miRNAs and diseases

To construct more accurate similarity related to miRNA and disease, we combined the Gaussian interaction spectral kernel similarity with the miRNA functional similarity and the disease semantic similarity. Based on previous study [14], the integrated similarity for miRNAs *IM*(*m*_*i*_, *m*_*j*_) and diseases *ID*(*d*_*i*_, *d*_*j*_) can be calculated as below:

$$\begin{array}{c} \begin{array}{cc} IM(m_{i},m_{j}) & =\{\begin{array}{cc} \frac{MFS\left(m_{i},m_{j}\right)+GPSM\left(m_{i},m_{j}\right)}{2}, & \text{if}\;m_{i}\;and\;m_{j}\;have\\ & functional\;similarity\\ GPSM(m_{i},m_{j}), & \text{otherwise}. \end{array} \end{array} \end{array}$$ (13)

$$\begin{array}{c} \begin{array}{cc} ID(d_{i},d_{j}) & =\{\begin{array}{cc} \frac{DSS\left(d_{i},d_{j}\right)+GPSD\left(d_{i},d_{j}\right)}{2}, & \text{if}\;d_{i}\;and\;d_{j}\;have\\ & semantic\;similarity\\ GPSD(d_{i},d_{j}), & \text{otherwise}. \end{array} \end{array} \end{array}$$ (14)

### HGCLAMIR

**Fig 1:**
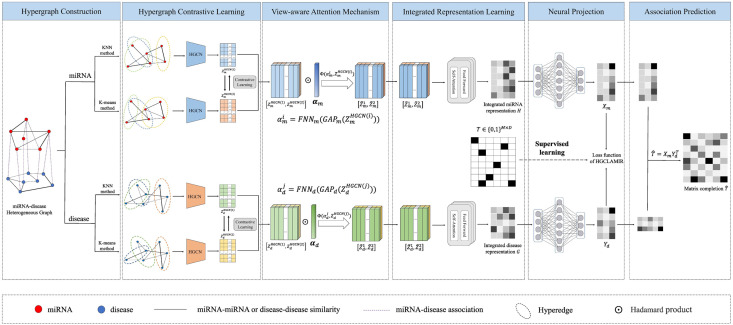
The workflow of our proposed HGCLAMIR model for predicting potential miRNA-disease associations.

In this paper, we proposed an end-to-end hypergraph contrastive learning with view-aware attention mechanism and integrated multi-view representation model for predicting the associations between miRNAs and diseases. As shown in Fig 1, HGCLAMIR model mainly includes hypergraph construction, hypergraph convolutional network (HGCN), hypergraph contrastive learning, view-aware attention mechanism, integrated representation learning and neural projection. More specifically, we first separately used KNN and K-means methods to construct hypergraphs related to miRNAs and diseases from the miRNA-disease heterogeneous graph. Then, we utilized HGCN to learn the miRNA (or disease) embedding representation of two different views. Furthermore, the hypergraph contrastive learning was proposed by combining HGCN with contrastive learning to improve and enhance the embedded representation learning ability of HGCN. Next, we introduced view-aware attention mechanism to obtain the importance of embedding representations of different views. Meanwhile, integrated representation learning was proposed to effectively integrate the enhanced embedding representations of different views. Finally, we input the integrated embedding representations into a neural network-based matrix completion method to identify miRNA-disease associations.

### Hypergraph construction and convolutional network

To capture beyond pairwise relationships from heterogeneous miRNA-disease networks, we adopted a weighted hypergraph *G* = (*V*, *E*, *W*) to represent a hyperedge related to miRNAs (or diseases). Concretely, $V=\{v_{1},v_{2},\ldots ,v_{\bar{m}}\}$ is the finite set of vertices of the hypergraph. $E=\{e_{1},e_{2},\ldots ,e_{\bar{n}}\}$ is the set of hyperedges of the hypergraph, and each hyperedge *e* is a subset of *V*. $W=\{W_{1},W_{2},\ldots ,W_{\bar{n}}\}$ represents the weight of hyperedge, which is a diagonal matrix. In this paper, we concatenated miRNA-disease associations and integrated miRNA similarity as features of node miRNAs. Similarly, we concatenated disease-miRNA associations and integrated disease similarity as features of node diseases. Based on the concatenated features, we utilized KNN and K-means methods to learn hypergraphs for miRNAs and diseases, respectively. To be more specific, we first calculated the nearest *k* neighbors of each miRNA based on Euclidean distance in the KNN method, thereby determining a subset (i.e., hyperedge) from *k* neighbors. The K-means method randomly selects the clustering center and uses Euclidean distance to determine the distance between each miRNA and the clustering center, thereby grouping those with close distances into one category and form a subset (i.e., hyperedge). Through multiple iterations until the clustering center no longer undergo changes. Then, the relationship between vertices and hyperedges in hypergraphs can be represented by the incidence matrix $H\in R^{\bar{m}\times \bar{n}}$. In a hypergraph constructed using the KNN method, the number of miRNAs is equal to the number of hyperedges, so the incidence matrix *H* is usually a square matrix. In a hypergraph constructed using the K-means method, the number of clustering center *c* is equal to the number of hyperedges, so the incidence matrix *H* is not necessarily a square matrix. Specifically, the element-wise representation of the incidence matrix *H* is defined as follows:

$$\begin{array}{c} H\left(v,e\right)=\{\begin{array}{cc} 1, & \text{if}\;v\in e\\ 0, & \text{otherwise}. \end{array} \end{array}$$ (15)

Based on previous study [37], hypergraph convolutional network (HGCN) using spectral convolution can well encode high-order relations in a hypergraph structure. According to the incidence matrix *H* and the weight *W* of the hyperedge, we can build a hyperedge convolution layer of HGCN as follows:

$$\begin{array}{c} X^{\left(l+1\right)}=\sigma \left(D_{v}^{-1/2}HWD_{e}^{-1}H^{T}D_{v}^{-1/2}X^{\left(l\right)}\Theta^{\left(l\right)}\right) \end{array}$$ (16)

where *X*^(*l*)^ is the aggregated information of hypergraph at *l* layer, *X*^(0)^ = *X*. Θ^(*l*)^ is the learnable filter matrix of the *l*-th layer. *σ*(⋅) denotes the nonlinear activation function. *D*_*e*_ and *D*_*v*_ represent the diagonal matrices of edge and vertex degrees, respectively. Concretely, the degree of a vertex *v* is defined as *d*(*v*) = ∑_*e*∈*E*_*w*(*e*)*H*(*v*, *e*). The degree of an edge *e* is defined as *d*(*e*) = ∑_*v*∈*V*_*H*(*v*, *e*).

### Hypergraph contrastive learning

**Fig 2:**
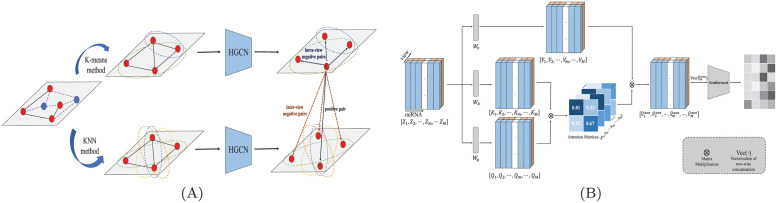
The detailed description of different modules in HGCLAMIR. (A) Illustration of hypergraph contrastive learning. (B) Introduction of integrated representation learning.

To improve and enhance the embedding quality in supervised learning, contrastive learning has become an effective solution [38, 39]. In recent years, many studies have also begun to combine contrastive learning and graph representation learning to enhance graph embedding representations [40, 41], which aim to learn good data representations by optimizing a contrastive loss generated from positive and negative pairs. Therefore, we proposed a hypergraph contrastive learning method to seek the consistency of the same node and the difference of different nodes in different views as shown in Fig 2A. Specifically, we first utilized KNN and K-means methods to construct two different hypergraph views, respectively. Then, we employed a contrastive objective function that enforces the encoded embeddings of each node in two different views to be consistent with each other and distinguishable from embeddings of other nodes. For any node *v*_*i*_, its embedding generated in one view, ***u***_*i*_, is regarded as the anchor, where the bold lowercase letters are used to represent vectors. The embedding of node *v*_*i*_ generated in the other view is represented as ***v***_*i*_. Next, the different embeddings ***u***_*i*_ and ***v***_*i*_ of the same node in two views form the positive sample, and are naturally regarded as negative samples with other embeddings ***u***_*k*_ and ***v***_*k*_ (*k* ≠ *i*) in two views. To be more specific, the embedding ***u***_*k*_ of other nodes in the same view as anchor ***u***_*i*_, which forms intra-view negative pairs with ***u***_*i*_. The embedding ***v***_*k*_ of other nodes is not in the same view as anchor ***u***_*i*_, which forms inter-view negative pairs with ***u***_*i*_. Similar to InfoNCE [42], we defined the pairwise training objective for each positive pair (***u***_*i*_, ***v***_*i*_) as follows:

$$\begin{array}{l} L_{CL}\left(u_{i},v_{i}\right)=\\ -\text{log}\frac{e^{\theta \left(u_{i},v_{i}\right)/\tau }}{\underset{\text{positive}\;\;\text{pair}}{\underset{︸}{e^{\theta \left(u_{i},v_{i}\right)/\tau }}}+\underset{\begin{array}{c} \text{intra-view}\\ \text{negative}\;\;\text{pairs} \end{array}}{\underset{︸}{\sum_{k\neq i}e^{\theta \left(u_{i},u_{k}\right)/\tau }}}+\underset{\begin{array}{c} \text{inter-view}\\ \text{negative}\;\;\text{pairs} \end{array}}{\underset{︸}{\sum_{k\neq i}e^{\theta \left(u_{i},v_{k}\right)/\tau }}}} \end{array}$$ (17)

where *τ* is a temperature parameter, the critic *θ*(***u***, ***v***) = *s*(*g*(***u***), *g*(***v***)). Here, *s*(⋅, ⋅) is the cosine similarity and *g*(⋅) is a nonlinear projection to enhance the expression power of the critic function [38]. In our method, the projection function *g* was implemented with a two-layer perceptron model.

In terms of *M* miRNAs, the contrastive loss of the hypergraph constructed by KNN can be defined as follows:

$$\begin{array}{c} \begin{array}{cc} & L_{CL}^{m1}\left(u_{i},v_{i}\right)=\\ & -\sum_{i=1}^{M}\text{log}\frac{e^{\theta (u_{i},v_{i})/\tau }}{e^{\theta (u_{i},v_{i})/\tau }+\sum_{k\neq i}e^{\theta (u_{i},u_{k})/\tau }+\sum_{k\neq i}e^{\theta (u_{i},v_{k})/\tau }} \end{array} \end{array}$$ (18)

Since the two views constructed by KNN and K-means for miRNAs are symmetric, we can obtain another contrastive loss of the hypergraph constructed by K-means which is also defined similarly as $L_{CL}^{m2}\left(v_{i},u_{i}\right)$.

Finally, we obtained the overall contrastive loss function for miRNAs as follows:

$$\begin{array}{c} L_{CL}^{m}=\eta L_{CL}^{m1}\left(u_{i},v_{i}\right)+\left(1-\eta \right)L_{CL}^{m2}\left(v_{i},u_{i}\right) \end{array}$$ (19)

where *η* is a coefficient to balance the effect of two views. In this paper, we set *η* to 0.5 for simplicity in our experiments. Similarly, we can obtain the overall contrastive loss function $L_{CL}^{d}$ for diseases.

### View-aware attention mechanism

In general, the quality of embedding representations of miRNAs (or diseases) varies between different views, resulting in different contributions to the prediction of miRNA-disease associations. To learn the importance of different views obtained by HGCN, we utilized global average pooling and fully connected neural network (FNN) to calculate attention weights of the *i*-th views for miRNAs as follows:

$$\begin{array}{c} \alpha_{m}^{i}=FNN_{m}\left(GAP_{m}\left(Z_{m}^{HGCN(i)}\right)\right) \end{array}$$ (20)

where *GAP*_*m*_(⋅) represents a global average pooling layer for miRNAs. *FNN*_*m*_(⋅) is a two-layer FNN for miRNAs, and the nonlinear activation functions of two layers are ReLU activation and Sigmoid activation, respectively. $Z_{m}^{HGCN(i)}$ denotes the embedding representation of the *i*-th view output by HGCN. Then, the two-view attention weight of miRNAs can be obtained as $\alpha_{m}=\left[\alpha_{m}^{1},\alpha_{m}^{2}\right]$.

Finally, we combined the embedding representations of different views with attention weights, which is defined as follows:

$$\begin{array}{c} {\tilde{Z}}_{m}^{i}=\Phi \left(\alpha_{m}^{i},Z_{m}^{HGCN(i)}\right)=\delta \left(\alpha_{m}^{i}\cdot Z_{m}^{HGCN(i)}\right) \end{array}$$ (21)

where *δ*(⋅) indicates ReLU activation function. Through the above steps, we can obtain the miRNA embedding representation information with attention weights ${\tilde{Z}}_{m}=\left[{\tilde{Z}}_{m}^{1},{\tilde{Z}}_{m}^{2}\right]$.

Similarly, the disease embedding representation information with attention weights for the *j*-th view is calculated as follows:

$$\begin{array}{c} \alpha_{d}^{j}=FNN_{d}\left(GAP_{d}\left(Z_{d}^{HGCN(j)}\right)\right) \end{array}$$ (22)

$$\begin{array}{c} {\tilde{Z}}_{d}^{j}=\Phi \left(\alpha_{d}^{j},Z_{d}^{HGCN(j)}\right)=\delta \left(\alpha_{d}^{j}\cdot Z_{d}^{HGCN(j)}\right) \end{array}$$ (23)

where *GAP*_*d*_(⋅) is a global average pooling layer for diseases. *FNN*_*d*_(⋅) is a two-layer FNN for diseases. The two-view attention weight of diseases can be obtained as $\alpha_{d}=\left[\alpha_{d}^{1},\alpha_{d}^{2}\right]$. Through the above steps, the final disease embedding representation information with attention weights can be defined as ${\tilde{Z}}_{d}=\left[{\tilde{Z}}_{d}^{1},{\tilde{Z}}_{d}^{2}\right]$.

### Integrated representation learning

After view-aware attention mechanism, we can obtain two miRNA (or disease) embedding information with attention weights from different perspectives. Inspired by Transformer encoder [43], we proposed integrated representation learning to integrate different views for achieving richer embedded representations in Fig 2B. For a miRNA *m*, we first concatenated the vectors ${\tilde{z}}_{m}^{1}$ and ${\tilde{z}}_{m}^{2}$ to obtain the embedding representation matrix of its two views as ${\hat{Z}}_{m}=\left[{\tilde{z}}_{m}^{1},{\tilde{z}}_{m}^{2}\right]$. Then, the query matrix $Q_{m}=W_{q}{\hat{Z}}_{m}=\left[q_{m}^{1},q_{m}^{2}\right]$, the key matrix $K_{m}=W_{k}{\hat{Z}}_{m}=\left[k_{m}^{1},k_{m}^{2}\right]$ and the value matrix $V_{m}=W_{v}{\hat{Z}}_{m}=\left[v_{m}^{1},v_{m}^{2}\right]$ can be obtained through the projection matrices *W*_*q*_, *W*_*k*_ and *W*_*v*_. Further, the scaled dot product function was chosen as the attention function [43]. Finally, the inter-view attention matrix *A*_*m*_ can be computed as follows:

$$\begin{array}{c} A_{m}\left(i,j\right)=\frac{\text{exp}[{\left(q_{m}^{i}\right)}^{T}\cdot k_{m}^{j}/\sqrt{d_{f}}]}{\sum_{j=1}^{2}\;\text{exp}\left[{\left(q_{m}^{i}\right)}^{T}\cdot k_{m}^{j}/\sqrt{d_{f}}\right]} \end{array}$$ (24)

where *A*_*m*_(*i*, *j*) represents how much concern the *i*-th view has for the *j*-th view of miRNA *m*, *d*_*f*_ refers to the dimension of the embedded representation for miRNAs. For two views, the inter-view attention matrix $A_{m}\left(i,j\right)\in R^{2\times 2}$ for a miRNA *m*. Note that we can obtain *M* inter-view attention matrices for *M* miRNAs. In addition, we considered inter-view attention, so the interaction between different views can be highlighted.

To improve the expressive ability and obtain the robust learning process, we extended self-attention to a multi-head version. Multi-head attention can be obtained by the following formulas:

$$\begin{array}{c} {\hat{V}}_{m}=A_{m}\cdot V_{m}^{T};\;{\hat{V}}_{m}^{ave}=\frac{1}{N}\sum_{p=1}^{N}{\left({\hat{V}}_{m}^{T}\right)}^{p} \end{array}$$ (25)

where *N* denotes the number of head. Moreover, different heads can capture different perspective information.

Finally, we utilized a two-layer Feedforward network to further encode the embedding representations obtained from multi-head attention. The detailed calculation formula is shown below:

$$\begin{array}{c} h_{m}=W_{h}\cdot \text{Vec}\left({\hat{V}}_{m}^{ave}\right) \end{array}$$ (26)

where *W*_*h*_ is used to represent parameters in the Feedforward network. Vec(⋅) represents the vectorization of row-wise concatenation. Then, the miRNA embedding representation matrix can be expressed as *H* = [***h***_1_, ***h***_2_, ⋯, ***h***_*m*_, ⋯, ***h***_*M*_] for *M* miRNAs. Similarly, the disease embedding representation matrix can be calculated as *G* = [***g***_1_, ***g***_2_, ⋯, ***g***_*d*_, ⋯, ***g***_*D*_] for *D* diseases.

### Optimization of HGCLAMIR

Through integrated representation learning, we obtained the integrated miRNA embedding representation *H* and the integrated disease embedding representation *G*, respectively. Based on the integrated representation information *H* and *G*, the neural network-based matrix completion method was utilized to perform miRNA-disease association prediction. More specifically, we used the fully connected neural network to obtain the final miRNA embedding representation matrix *X*_*m*_ and disease embedding representation matrix *Y*_*d*_. Then, we obtained the reconstructed association matrix $\hat{T}$ by matrix multiplication as shown below:

$$\begin{array}{c} \hat{T}=X_{m}Y_{d}^{T} \end{array}$$ (27)

There is an imbalance problem that unknown (or unobserved) is much larger than observed in the association matrix between miRNAs and diseases, which will affect the training of the model. To alleviate this problem, we introduced a tradeoff parameter *α* to balance the observed and unknown (or unobserved) entries well. Finally, the objective function of our model can be more accurately defined as follows:

$$\begin{array}{c} L_{RE}=\frac{\left(1-\alpha \right)}{2}∥P_{\Omega }\left(T-\hat{T}\right){∥}_{F}^{2}+\frac{\alpha }{2}{∥P_{\bar{\Omega }}\left(T-\hat{T}\right)∥}_{F}^{2} \end{array}$$ (28)

where Ω and $\bar{\Omega }$ represent the set of observed, unobserved or unknown miRNA-disease entries from the known association matrix *T*, respectively.

Finally, the optimization objective of our model consists of three parts: the reconstruction loss, the contrastive loss for miRNAs and the contrastive loss for diseases:

$$\begin{array}{c} L=L_{RE}+\lambda L_{CL}^{m}+\gamma L_{CL}^{d} \end{array}$$ (29)

where λ and *γ* control the impact of contrastive loss for miRNAs $L_{CL}^{m}$ and diseases $L_{CL}^{d}$, respectively. In this paper, we set λ and *γ* to 1 for simplicity. Meanwhile, we used Adam [44] with learning rate *β* to optimize the HGCLAMIR model based on PyTorch.

## Results

### Implementation details and evaluation metrics

Based on previous work [45], we randomly selected 9/10 samples from a sample set containing all positive and negative samples to generate the cross-validation set, and utilized the remaining 1/10 samples as the independent test set. Note that there is no overlap between the cross-validation set and the independent test set. In this paper, we performed cross-validation experiments and parameter analysis by conducting the 5-fold cross-validation on the cross-validation set. To be more specific, all experimentally verified miRNA-disease associations were randomly divided into five equal subsets. In each fold, one subset as testing set in turn and the other four subsets as training sets. Meanwhile, in order to make a more reasonable and fair performance analysis, we compared the proposed model with other baseline models on an independent test set. The area under the precision-recall (AUPR) curve, the area under the receiver operating characteristic (AUC) curve and F1 score were used to evaluate the prediction performance of all models.

### Baseline models

To comprehensively evaluate the prediction performance of our proposed HGCLAMIR model, we introduced the following several models as baselines.

IMCMDA [34]: IMCMDA utilizes the inductive matrix completion method for miRNA-disease association prediction based on integrated miRNA and disease similarity matrices.

PBMDA [13]: The miRNA-disease associations, integrated miRNA and disease similarity information are used to construct a heterogeneous graph. Then, PBMDA applies a depth-first search algorithm to infer potential associations between miRNAs and diseases based on the heterogeneous graph.

GRGMF [46]: Zhang *et al*. developed a graph regularized generalized matrix factorization method to infer potential associations in biomedical bipartite networks.

NIMCGCN [30]: The method aggregates the embedded information of miRNAs and diseases by utilizing GCN and applies a neural inductive matrix completion method to infer miRNA-disease associations.

MMGCN [23]: MMGCN performs GCN to capture the embedded representation of multi-view miRNA and disease and uses the attention mechanism to learn the importance of different views. Then, CNN is utilized to integrate multi-view embedded information for predicting potential miRNA-disease associations.

MvKFN-MDA [47]: Multiple kernel fusion network is used to integrate the similarity information of multi-view miRNA and disease. Then, these integrated similarities are fed to a neural matrix completion method to infer the potential associations between miRNAs and diseases.

GCAEMDA [48]: GCAEMDA uses graph convolutional autoencoder to learn scores of miRNA-disease from miRNA-based and disease-based sub-networks, and adopts an average ensemble way to integrate two prediction scores for the final miRNA-disease association prediction.

MSGCL [26]: The method employs self-supervised contrastive learning to optimize the graph structure and utilizes a graph convolutional network encoder to infer the associations between miRNAs and diseases.

ERMDA [49]: Dai *et al*. proposed an ensemble learning framework with resampling method for miRNA-disease association (ERMDA) prediction to discover potential disease-related miRNAs.

AMHMDA [29]: AMHMDA method, leveraging attention aware multi-view similarity networks and hypergraph learning, introduces hypernodes into the graph convolution network to learn high-quality links and richer node information for miRNA-disease association identification.

### Parameters analysis

**Fig 3:**
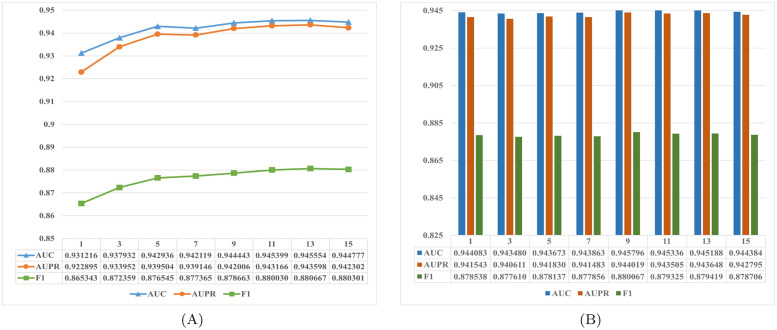
The influence of different hyperparameters on HGCLAMIR based on the MDAv2.0 dataset under 5-fold cross-validation. (A) The impact of hyperparameter *k* on HGCLAMIR. (B) The impact of hyperparameter *c* on HGCLAMIR.

In this section, we showed the influence of several hyperparameters on the performance of HGCLAMIR on the MDAv2.0 dataset. Furthermore, we used cross-validation and AUC, AUPR, F1 values to evaluate them for selecting the optimal hyperparameters. Among them, the hyperparameters mainly include *k* in KNN method, the number of clustering center *c* in K-means method, learning rate *β* and the biased item *α* in the loss function defined by Eq 28. First, we fixed the other hyperparameters to select the optimal *k* value. Specifically, we searched the optimal *k* value from {1, 3, 5, ⋯, 13, 15}. As shown in Fig 3A, we found that when *k* was set to 13, HGCLAMIR model obtained the optimal prediction performance. In a similar way, optimal *c* value can be found from {1, 3, 5, ⋯, 13, 15} and set *c* = 9 in Fig 3B. Then, training *α* within {0.01, 0.03, 0.05, ⋯, 0.13, 0.15} and set *α* = 0.11 in S1(A) Fig. Finally, we searched the optimal *β* from {0.00001, 0.0001, 0.001, 0.01, 0.1} and set *β* = 0.0001 in S1(B) Fig. It is worth noting that other experimental datasets also require hyperparameter selection, and detailed hyperparameter adjustment results can be obtained in S2 Fig.

### Comparison experiments

For a fairer comparative analysis with other baseline models, all comparison experiments were conducted with the same datasets and experimental settings. First, we compared HGCLAMIR with other models by performing 5-fold cross-validation five times on cross-validation sets. Moreover, when evaluating the prediction performance of the model, we also randomly selected unobserved elements equal to the positive sample size as negative samples 10 times and reported the average results to obtain a more reasonable evaluation. As shown in Table 1, our proposed HGCLAMIR model achieved the best prediction performance on all datasets.

**Table 1:** The prediction performance of all models evaluated by 5-fold cross-validation five times.

Model	MDAv2.0			MDAv3.2		
	AUC	AUPR	F1	AUC	AUPR	F1
IMCMDA	0.848512	0.868595	0.778759	0.878090	0.887196	0.811497
PBMDA	0.916204	0.920863	0.846252	0.935506	0.929664	0.867088
GRGMF	0.921368	0.932778	0.857465	0.938011	0.946759	0.877490
NIMCGCN	0.936045	0.935408	0.866438	0.954876	0.953186	0.892786
MMGCN	0.917045	0.933986	0.864688	0.937804	0.946449	0.888515
MvKFN-MDA	0.937915	0.936040	0.870066	0.958430	0.955316	0.895589
GCAEMDA	0.930890	0.943125	0.874576	0.933799	0.946583	0.876278
MSGCL	0.906431	0.912735	0.846619	0.931279	0.934223	0.874771
ERMDA	0.938933	0.937784	0.864552	0.959165	0.954715	0.893585
AMHMDA	0.923040	0.919820	0.835820	0.951680	0.946420	0.876040
HGCLAMIR	**0.945284**	**0.945074**	**0.879973**	**0.962600**	**0.959563**	**0.902512**

**Fig 4:**
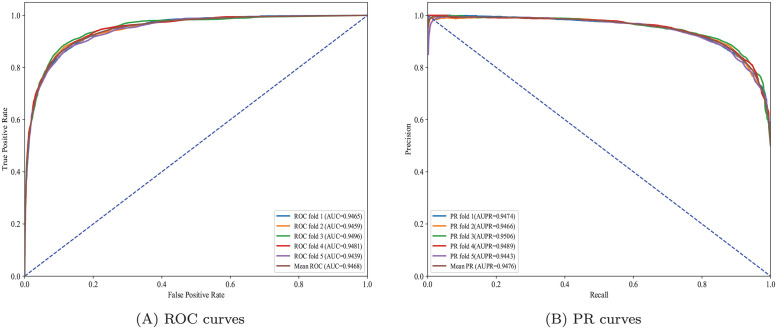
ROC curves and PR curves performed by HGCLAMIR based on the MDAv2.0 dataset under 5-fold cross-validation.

**Fig 5:**
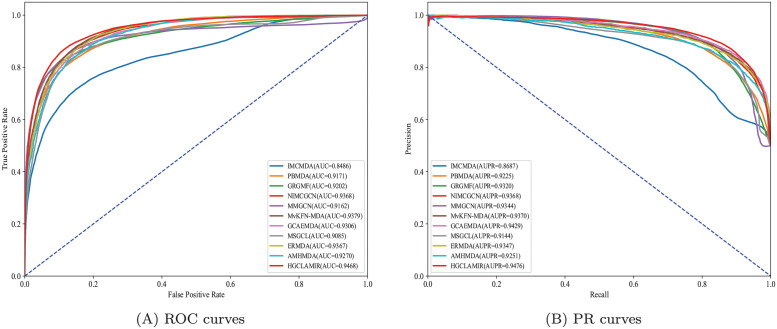
ROC curves and PR curves performed by HGCLAMIR and ten baseline models based on the MDAv2.0 dataset under 5-fold cross-validation.

To be more specific, the average AUC value of 5-fold cross-validation five times of HGCLAMIR on the MDAv2.0 dataset is 0.945284, whereas the average AUC values of IMCMDA, PBMDA, GRGMF, NIMCGCN, MMGCN, MvKFN-MDA, GCAEMDA, MSGCL, ERMDA and AMHMDA are 0.848512, 0.916204, 0.921368, 0.936045, 0.917045, 0.937915, 0.930890, 0.906431, 0.938933, 0.923040, respectively. Similarly, HGCLAMIR model was also significantly better than ten comparison models on the MDAv3.2 dataset. It is worth noting that the prediction performance of HGCLAMIR can open up a significant gap compared with IMCMDA, PBMDA and GRGMF. This may be due to the fact that HGCLAMIR using hypergraph convolutional network can better capture complex nonlinear relationships in biological heterogeneous networks, thereby improving prediction performance. Then, we more intuitively displayed the prediction performance of the proposed HGCLAMIR model in graphical form based on MDAv2.0 and MDAv3.2 datasets. From Figs 4 and S3, we observed that the prediction performance of HGCLAMIR in each fold is not much different under 5-fold cross-validation, which further indicated that the performance of HGCLAMIR is relatively stable. In addition, Figs 5 and S4 show the comparative ROC curves and PR curves performed by HGCLAMIR and ten baseline models under 5-fold cross-validation, from which we can see that HGCLAMIR still achieved better prediction performance on MDAv2.0 and MDAv3.2 datasets. Finally, in order to stricter evaluate the prediction performance of HGCLAMIR, we further considered the issue of avoiding data leakage based on previous study [50]. More specifically, the training set’s known associations were used to calculate biological similarities related to miRNAs and diseases. From S1 Table, we observed that the prediction performance of HGCLAMIR is still better than other baseline models, without a huge drop in performance. This further demonstrates that the HGCLAMIR model has good robustness and can be considered as an effective tool to predict miRNA-disease associations.

To avoid over-optimistic results on cross-validation set, we further conducted comparative analysis of all models on independent test set. More specifically, all models were trained on the cross-validation set and performed miRNA-disease association prediction on an independent test set. Since the model parameters were selected by 5-fold cross-validation on the cross-validation set and training is irrelevant to independent test set, we can evaluate the prediction performance of all models on the independent test set for unseen data. Table 2 shows the prediction performance of all models on an independent validation set, from which we can see that HGCLAMIR also achieved the best prediction results in terms of AUC, AUPR and F1 on all datasets. This result demonstrated that our model has better generalization ability.

**Table 2:** The prediction performance of all models based on an independent dataset.

Model	MDAv2.0			MDAv3.2		
	AUC	AUPR	F1	AUC	AUPR	F1
IMCMDA	0.820052	0.833042	0.758093	0.858128	0.861453	0.794376
PBMDA	0.912210	0.911953	0.840912	0.934865	0.931506	0.866340
GRGMF	0.929045	0.937174	0.862512	0.943598	0.949517	0.883890
NIMCGCN	0.926954	0.923480	0.855727	0.950184	0.948709	0.887152
MMGCN	0.907686	0.924860	0.852278	0.931711	0.944657	0.884877
MvKFN-MDA	0.927787	0.924527	0.855139	0.954336	0.951087	0.892343
GCAEMDA	0.923456	0.925235	0.855872	0.922735	0.925077	0.853783
MSGCL	0.902129	0.906020	0.845174	0.930606	0.933127	0.880590
ERMDA	0.931979	0.927496	0.861035	0.954571	0.949297	0.883530
AMHMDA	0.909000	0.910900	0.823900	0.943100	0.935600	0.863900
HGCLAMIR	**0.935995**	**0.937769**	**0.872038**	**0.956750**	**0.953737**	**0.894457**

### Ablation studies

To better verify the effectiveness of hypergraph convolutional network (HGCN), contrastive learning, view-aware attention mechanism and integrated representation learning, we constructed GCN_AMIR, HGCN_AMIR, HGCLAM_concat and HGCL_IR as four variants of HGCLAMIR for comparative analysis. (1) GCN_AMIR: we replaced HGCN with GCN to explore the ability of hypergraph convolutional network to capture complex high-order relations. To obtain embedding information of two different views for miRNAs (or diseases), we utilized GCN to extract embedding representations of different views of miRNAs (or diseases) from two different biological similarity information. (2) HGCN_AMIR: we removed the contrastive learning and kept other modules unchanged to explore whether the contrastive learning can enhance the embedding representation learning ability of HGCN, thereby improving the prediction performance of the model. (3) HGCLAM_concat: in order to verify the ability of integrated representation learning to integrate multi-view embedding information, we retained other modules except integrated representation learning. (4) HGCL_IR: we only removed view-aware attention mechanism to explore whether paying attention to the importance of different views can effectively improve model prediction performance.

Table 3 shows the prediction performance of different variants evaluated by 5-fold cross-validation five times on MDAv2.0 and MDAv3.2 datasets. From Table 3, we can see that the prediction performance of HGCN_AMIR was significantly better than that of GCN_AMIR. This result demonstrated that compared with GCN, HGCN can better capture complex high-order relations in heterogeneous graphs, thereby learning high-quality embedding representations. Moreover, after using integrated representation learning, HGCLAMIR obtained better prediction performance than HGCLAM_concat, which suggested that integrated representation learning can learn richer embedding information and integrate it reasonably. Similarly, after using view-aware attention mechanism, HGCLAMIR also achieved better prediction performance than HGCL_IR, which showed that the introduction of view-aware attention mechanism can improve prediction performance by adaptively weighting the embedded representations of different views. In addition, compared with HGCLAMIR, the prediction performance of HGCN_AMIR had a certain decline, indicating that contrastive learning can enhance the learning ability of embedded representation of HGCN, thereby contributing to improving model prediction performance.

**Table 3:** The prediction performance of ablation experiment evaluated by 5-fold cross-validation five times.

		AUC	AUPR	F1
MDAv2.0	GCN_AMIR	0.938786	0.938487	0.870857
	HGCN_AMIR	0.943115	0.942972	0.878551
	HGCLAM_concat	0.940708	0.939349	0.873361
	HGCL_IR	0.942953	0.942833	0.877939
	HGCLAMIR	**0.945284**	**0.945074**	**0.879973**
MDAv3.2	GCN_AMIR	0.958718	0.956102	0.895865
	HGCN_AMIR	0.961492	0.958568	0.901617
	HGCLAM_concat	0.960346	0.958133	0.899518
	HGCL_IR	0.961823	0.959096	0.901406
	HGCLAMIR	**0.962600**	**0.959563**	**0.902512**

### Case studies

To further verify the accuracy of HGCLAMIR in predicting the associations between miRNAs and specific diseases, we performed case studies on two important tumor diseases, namely breast neoplasms and lung neoplasms, based on the MDAv2.0 dataset. More specifically, we utilized negative miRNA-disease associations and experimentally verified positive miRNA-disease associations to construct training samples, which excluded the specific disease for case studies. Then, the associations between miRNAs and the specific disease were used to construct testing samples. Finally, we trained HGCLAMIR model on training samples, and used the trained model to predict the associations between miRNAs and the specific disease. In addition, we ranked the predicted results and selected the top prediction scores as the candidates. Meanwhile, we verified the top 50 prediction results by finding supporting evidence according to the lasted HMDD v4.0 [51] and dbDEMC [52].

Table 4 shows the prediction and verification results of miRNAs related to breast neoplasms. From Table 4, we can see that 49 of the top 50 predicted breast neoplasms-related miRNAs were successfully confirmed by HMDD v4.0 and dbDEMC databases, whereas the miRNAs that were not confirmed by the relevant databases were marked as “unconfirmed”. Similarly, the prediction and verification results of lung neoplasms-related miRNAs are shown in S2 Table. The 48 of the top 50 predicted lung neoplasms-related miRNAs were verified with the above two databases. At the same time, we also observed that these miRNAs with higher similarity were predicted to be associated with the same specific disease. For example, hsa-mir-130a and hsa-mir-130b with high similarity are closely related to the occurrence and development of breast cancer [53, 54], which further confirmed the necessity of integrating biological similarity networks.

**Table 4:** Top 50 breast neoplasms-related miRNAs predicted by HGCLAMIR based on the MDAv2.0 dataset. Note that the number in evidence means PubMed Unique Identifier (PMID).

Rank	miRNA	Score	Evidence	Rank	miRNA	Score	Evidence
1	hsa-mir-142	1.15441	25406066	26	hsa-mir-361	1.00183	36622663
2	hsa-mir-378a	1.09240	26255816	27	hsa-mir-28	1.00172	34593318
3	hsa-mir-15b	1.08877	22908280	28	hsa-mir-32	0.99690	29661250
4	hsa-mir-372	1.07984	29456685	29	hsa-mir-498	0.99468	35715772
5	hsa-mir-190a	1.07910	24009311	30	hsa-mir-508	0.99141	36161346
6	hsa-mir-150	1.07259	25907662	31	hsa-mir-216a	0.98864	32916503
7	hsa-mir-217	1.06926	36357766	32	hsa-mir-1224	0.98777	33986801
8	hsa-mir-30e	1.06789	25523096	33	hsa-mir-502	0.98282	27080302
9	hsa-mir-138	1.04343	27155849	34	hsa-mir-494	0.98012	27216190
10	hsa-mir-532	1.04181	36077054	35	hsa-mir-211	0.97953	35296964
11	hsa-mir-330	1.04007	dbDEMC	36	hsa-mir-449b	0.97269	32374522
12	hsa-mir-130b	1.03679	26152113	37	hsa-mir-491	0.97098	25725194
13	hsa-mir-106a	1.03399	25883093	38	hsa-mir-542	0.97085	24846313
14	hsa-mir-130a	1.03038	25755726	39	hsa-mir-503	0.96584	29164842
15	hsa-mir-370	1.02825	25451164	40	hsa-mir-95	0.95705	dbDEMC
16	hsa-mir-185	1.02717	24846313	41	hsa-mir-212	0.95291	26377202
17	hsa-mir-192	1.02709	26642352	42	hsa-mir-362	0.95235	33962174
18	hsa-mir-517a	1.02612	dbDEMC	43	hsa-mir-520e	0.95187	31934637
19	hsa-mir-650	1.02087	33086498	44	hsa-mir-208a	0.94627	26460550
20	hsa-mir-186	1.02065	35351581	45	hsa-mir-216b	0.94386	25078617
21	hsa-mir-92b	1.01590	29661250	46	hsa-mir-198	0.93571	26152113
22	hsa-mir-512	1.01576	34873163	47	hsa-mir-134	0.93462	36340453
23	hsa-mir-371a	1.01192	unconfirmed	48	hsa-mir-485	0.93136	25003827
24	hsa-mir-99a	1.01144	25388283	49	hsa-mir-98	0.93056	24696733
25	hsa-mir-1249	1.00591	31097355	50	hsa-mir-513b	0.92721	34738869

**Fig 6:**
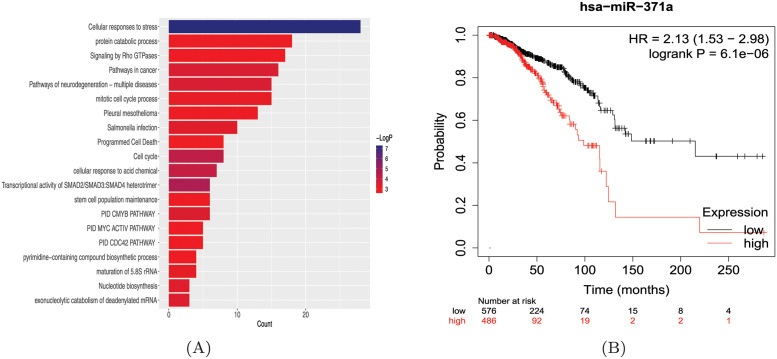
The biological analysis of hsa-mir-371a associated with breast neoplasms. (A) The enrichment analysis of target gene sets related to hsa-mir-371a. (B) The survival analysis based on hsa-mir-371a expression levels.

To further validate the biological significance of the potential miRNA-disease associations uncovered by the HGCLAMIR model, we performed enrichment analysis on gene sets consisting of specific miRNA target genes and survival analysis for disease-related candidate miRNAs. First, we obtained the target genes of miRNA from miRTarBase [55] and used Metascape [56] to explore which biological processes and pathway information are closely related to these target gene sets. From Fig 6A, it can be seen that the target gene set related to hsa-mir-371a was significantly enriched in several terms closely related to breast cancer, including Transcriptional activity of SMAD2/SMAD3:SMAD4 heterotrimer, pathways in cancer, mitotic cell cycle process, and Signaling by Rho GTPases. For example, Transcriptional activity of SMAD2/SMAD3:SMAD4 heterotrimer involved in the degradation of SKI/SKIL, thus causing malignant transformation in breast cancer [57]. The deregulation of cell cycle is a hallmark of cancer including breast cancer, which allows for limitless cell division [58, 59]. Studies have shown that Rho GTPases and their signaling components are overexpressed and/or are hyperactive in breast cancer, and that Rho GTPases are required for breast cancer cell metastasis in vivo [60]. Furthermore, we obtained many term information such as biological processes and pathways through the above enrichment analysis. To further capture the relationship between these terms, we performed cluster analysis using Metascape and selected the term with the best p-value to represent the cluster. As shown in S5 Fig, we found that several terms related to breast cancer were all statistically significant (p<0.01) and clustered together. Finally, we conducted survival analysis utilizing the miRpower-Kaplan-Meier plotter web-tool [61] to demonstrate the impact of hsa-mir-371a expression levels on the overall survival time of breast cancer patients. From Fig 6B, we observed that the expression level of hsa-mir-371a significantly affects the survival time of breast cancer patients, which further indicated that hsa-mir-371a may be involved in the development of breast cancer. To sum up, the results of the above biological analysis suggested that hsa-mir-371a may lead to the occurrence and development of breast cancer.

## Discussion and conclusion

Computational methods serve as effective complementary tools to traditional wet experiments in identifying potential miRNA-disease associations, which could improve our understanding of disease pathogenesis and accelerate the discovery of important biomarkers. In this study, we developed an HGCLAMIR model of hypergraph contrastive learning with view-aware attention mechanism and integrated multi-view representation for miRNA-disease association prediction. HGCLAMIR utilized hypergraph convolutional network to capture high-order complex relations in heterogeneous networks. To improve and enhance the embedded representation learning ability of HGCN, we combined HGCN with contrastive learning to learn higher quality embedding representations. Furthermore, view-aware attention mechanism was introduced to further improve prediction performance by adaptively weighting the embedding representations of different views. In addition, integrated representation learning was implemented to integrate the embedding representations of different views to obtain more reasonable embedding information. The experimental results of 5-fold cross-validation five times and independent validation showed that HGCLAMIR obtained better prediction performance and robustness than ten baseline models. Moreover, the results of the ablation experiment further demonstrated that the introduction of hypergraph convolutional network, contrastive learning, view-aware attention mechanism and integrated representation learning can effectively improve the prediction performance of the model. Meanwhile, the results of case studies indicated that 49 and 48 of the top 50 predicted disease-related miRNAs were verified by using published experimental studies, which showed that the HGCLAMIR model can accurately predict miRNA-disease associations. Furthermore, unconfirmed miRNA-disease associations had biological significance. To sum up, these results suggested that HGCLAMIR can be considered as an effective model for identifying potential miRNA-disease associations.

## Supporting information

S1 Fig The influence of different hyperparameters on HGCLAMIR based on the MDAv2.0 dataset under 5-fold cross-validation. (EPS)

S2 Fig The influence of different hyperparameters on HGCLAMIR based on the MDAv3.2 dataset under 5-fold cross-validation. (EPS)

S3 Fig ROC curves and PR curves performed by HGCLAMIR based on the MDAv3.2 dataset under 5-fold cross-validation. (EPS)

S4 Fig ROC curves and PR curves performed by HGCLAMIR and ten baseline models based on the MDAv3.2 dataset under 5-fold cross-validation. (EPS)

S5 Fig Network of enriched terms, where nodes that share the same cluster ID are typically close to each other. (EPS)

S1 Table The prediction performance of all models in considering the issue of avoiding data leakage. (XLSX)

S2 Table Top 50 lung neoplasms-related miRNAs predicted by HGCLAMIR based on the MDAv2.0 dataset. (XLSX)
